# The quality of dietary carbohydrate and fat is associated with better metabolic control in persons with type 1 and type 2 diabetes

**DOI:** 10.1186/s12937-020-00645-6

**Published:** 2020-11-19

**Authors:** Sabine S. Jacobsen, Dorte Vistisen, Tina Vilsbøll, Jens M. Bruun, Bettina Ewers

**Affiliations:** 1 Steno Diabetes Center Copenhagen, Niels Steensens Vej 4, DK-2820 Gentofte, Denmark; 2 Department of Clinical Medicine, Faculty of Health and Medical Sciences, University of Copenhagen, Copenhagen, Denmark; 3 Steno Diabetes Center Aarhus, Aarhus, Denmark; 4 Department of Clinical Medicine, University of Aarhus, Aarhus, Denmark; 5 Department of Nutrition, Exercise and Sports, University of Copenhagen, Frederiksberg, Denmark

**Keywords:** Nutrition, Dietary intake, Clinical markers, Carbohydrate quality, Type 1 diabetes, Type 2 diabetes

## Abstract

**Background:**

Diet quality is generally poor in persons with diabetes and it is unknown whether this is associated with worse glycaemic control and atherogenic lipid profile. The aim was to examine diet quality in relation to important markers of metabolic control in adults with type 1 diabetes (T1D) and type 2 diabetes (T2D).

**Methods:**

The study was cross-sectional and included 423 (49% females) persons with T1D and 339 (29% females) persons with T2D recruited from an outpatient diabetes clinic in Denmark. Data were collected from July 2014 to January 2015. Diet quality was assessed with a food frequency questionnaire to examine eight key dietary components (carbohydrates, saturated fatty acids, monounsaturated fatty acids, polyunsaturated fatty acids, added sugar, dietary fibre, fruit and vegetables). Clinical data assessing metabolic control (haemoglobin A1c (HbA1c), total cholesterol (total C), low-density lipoprotein cholesterol (LDL-C), high-density lipoprotein cholesterol (HDL-C), systolic and diastolic blood pressure and body mass index were extracted from the electronic medical records.

**Results:**

In T1D, higher intake of carbohydrates and added sugar was associated with higher HbA1c; higher fruit intake was associated with lower total C and LDL-C; and higher intake of carbohydrates and dietary fibre was associated with lower HDL-C. In T2D, higher intake of saturated fat was associated with higher total C; higher intake of added sugar was associated with higher LDL-C; and higher intake of polyunsaturated fat was associated with higher diastolic blood pressure.

**Conclusions:**

In Danish adults with T1D and T2D, both the total intake and the quality of carbohydrates and fat were associated with an unfavourable glucose regulation and lipid profile. Thus, our findings support a constant focus on diet and emphasise the need for dietary support in people with diabetes to improve diet quality, metabolic control and possibly reduce cardiovascular risk.

## Background

Dietary guidance for persons with type 1 (T1D) and type 2 diabetes (T2D) is based on systematic evidence-based dietary recommendations aiming to improve metabolic control and potentially reduce long-term diabetes-related complications [1–3]. This is accomplished by attaining and maintaining optimal glycaemic control (e.g. assessed by glycated haemoglobin A1c (HbA1c)), an acceptable body mass index (BMI) as well as optimal blood pressure and lipid levels to reduce the risk of micro- and macrovascular complications including cardiovascular disease (CVD) and mortality [3–5]. Despite these ambitions, diet quality and adherence to the dietary recommendations are generally poor in persons with T1D and T2D as indicated by a high consumption of saturated fat and low consumption of dietary fibre, vegetables, fruit and fish [6, 7]. In addition to the focus on the overall diet composition in persons with diabetes, carbohydrate intake is receiving growing attention in both research and clinical work [6, 8, 9]. Both the type and amount of carbohydrates in the diet has been proven critical for glycaemic control in persons with diabetes [2, 7, 10–15]. However, it is unclear whether a poor diet in relation to carbohydrate quality is associated with a more atherogenic lipid profile in these persons [6, 8]. Since diabetes is associated with an increased risk of developing CVD [16, 17] it is of major clinical interest to investigate associations between diet and health outcomes in persons with diabetes. Accordingly, we investigated eight key dietary indicators in relation to the four most commonly used clinical markers — HbA1c, lipid profile, blood pressure and BMI — in a large sample of adults with T1D and T2D.

## Methods

### Design and participants

The study was a cross-sectional design with all data collected during July 2014–January 2015. A random sample of 3000 adults (> 18 years) with diabetes (1500 with T1D and 1500 with T2D) followed in the outpatient clinic at Steno Diabetes Center Copenhagen were invited to the survey. Participants received an invitation with information regarding the questionnaire, including a personal token and a hyperlink to a website containing the study questionnaire. The online survey tool, Lime Survey (San Francisco, CA, USA), was connected to a server at the National Food Institute (Technical University of Denmark). Participants were offered a telephone hotline if in need of assistance with the questionnaire. The phone was answered by a dietitian. Information on the participants’ habitual diet, level of physical activity and level of education was collected from the self-administered web-based questionnaire. Questions and classification of level of education are according to Statistics Denmark (www.dst.dk/en). The Danish version of the International Physical Activity Questionnaire short form (IPAQ-SF) was used to collect data concerning the level of physical activity for the previous 7 days. Clinical data were collected from the participants’ electronic medical records (EMR) [7].

We included persons with diabetes-related complications that could influence dietary intake (e.g. gastropareses, coeliac disease and kidney disease); however, the number was small (< 5%). Exclusion criteria included mental disorders or life-threatening disorders. A total of 774 persons with diabetes responded to the invitation and participated in the study, from which we excluded 12 persons due to missing clinical data, leaving 762 study participants with complete data for analyses (*n* = 423 with T1D, *n* = 339 with T2D). Study flow chart, response rates, representativeness and a detailed description of the web-based survey tool have been described previously [7]. Participants were informed that completing the web-based questionnaire was regarded as consent to participate in the study according to Danish regulations for biomedical research. The dietary survey was approved *by the local ethics committee and the Danish Data Protection Agency.*

### Dietary data

Eight dietary indicators were chosen according to the latest American and European nutrition guidelines in diabetes, primarily with grade A and B evidence [1, 18].

Dietary intake of total energy (kJ/d), six nutrients (carbohydrates, saturated fatty acids (SFA), monounsaturated fatty acids (MUFA), polyunsaturated fatty acids (PUFA), added sugar and dietary fibre), and two healthy food groups (fruit and vegetables), all in gram/d were assessed using a validated web-based food frequency questionnaire (FFQ) designed to cover the food intake of the previous 3 months [9]. Intakes were calculated from the Danish Food Composition Database using standard portion sizes and recipes. The FFQ consists of 270 food items and mixed dishes. Portion sizes were estimated using the same household measures and series of photographs that participants could select according to their habitual dietary intake similar to the food diary in Danish National Survey of Dietary Habits and Physical Activity as previously described [7].

### Clinical data

Clinical data extracted from the EMR included age, sex, type of diabetes, diabetes duration, height and weight (used for calculation of BMI), smoking habits, plasma HbA1c, plasma levels of total cholesterol (total C), high-density lipoprotein cholesterol (HDL-C), and low-density lipoprotein cholesterol (LDL-C), diastolic (DBP) and systolic blood pressure (SBP), use of insulin pump, use of insulin, oral antidiabetic drugs (OAD) and lipid lowering medications. Blood samples were analysed for lipids with standard hospital methodology. The clinical values closest to the date of the completed web-based questionnaire were used.

### Statistical analyses

Analyses included standard descriptive statistics. All data are presented as medians with interquartile range (IQR). Associations between dietary components (carbohydrates, SFA, MUFA, PUFA, added sugar, dietary fibre, fruit and vegetables) and the clinical markers (HbA1c, BMI, total C, HDL-C, LDL-C, DBP and SBP) were analysed using linear regression models. Prior to analyses, we log-transformed exposure variables to ensure that model residuals were normally distributed. To further allow direct comparisons of the associations between the exposures (dietary components) for a given outcome (clinical marker) we standardized the dietary component variables prior to analyses. Standardization was done by dividing the log-transformed exposure variable by the standard deviation (SD) of the log-transformed values in the study population [19]. Associations between dietary intake and clinical markers were examined by multiple linear regression analysis and separately for persons with T1D and T2D with adjustment for relevant confounders in a stepwise approach. Model 1 was adjusted for sex, age, duration of diabetes, physical activity, smoking; Model 2 was additionally adjusted for BMI; Model 3 was additionally adjusted for education level and total energy. Furthermore, analyses of plasma HbA1c were additionally adjusted for insulin dose and use of pump (in persons with T1D) and insulin dose and OAD (in persons with T2D), analyses of plasma cholesterols were additionally adjusted for use of lipid-lowering medication and analyses of blood pressure were adjusted for blood pressure-lowering medication. For all statistical tests, a two-sided significance level of 0.05 was used. All statistical analyses were performed with the SPSS software for Windows, version 22.0 (IBM Corp, Armonk, NY, USA).

## Results

Participants characteristics are shown in Table 1. The group with T1D had equal numbers of male and female participants, and in the group with T2D, 71% were male participants. Median age was 53 years (41–64) among participants with T1D, and 65 years (58–71) among participants with T2D. Median HbA1c and lipid profile were close to treatment targets in both groups. Of the participants with T2D, 85–92% were on lipid-lowering and blood pressure-lowering medication; this applied to approximately half of those with T1D. More than 80% with T2D were treated with different OAD, 30% with glucagon-like peptide-1 receptor analogues (GLP-1a), and 66% with insulin.

**Table 1:** Baseline characteristics of study participants (*n* = 762)

Characteristics^a^	Participants T1D (*n* = 423)	Participants T2D (*n* = 339)
Sex (F/M), % (n)	49/51 (206/217)	29/71 (100/239)
Age, years	53 (41–64)	65 (58–71)
BMI, kg/m^2^	24.9 (22.6–27.6)	29.2 (26.5–33.3)
Insulin pump, % (n)	28.6 (121)	0 (0)
Years with diabetes, y	26 (14–39)	15 (9–22)
Smokers, % (n)	14 (58)	12 (39)
HbA1c, mmol/mol/ %	58 (52–65) / 7.5(6.9–8.1)	57 (51–66) / 7.3 (6.8–8.1)
Blood cholesterol levels		
Total cholesterol, mmol/l	4.5 (4.0–5.1)	4.0 (3.5–4.7)
HDL C, mmol/l	1.55 (1.30–1.94)	1.07 (0.89–1.30)
LDL C, mmol/l	2.5 (2.0–2.9)	2.0 (1.5–2.4)
Systolic blood pressure, mm Hg	128 (120–136)	130 (122–139)
Diastolic blood pressure, mm Hg	76 (70–82)	77 (71–82)
Medical treatment (%)		
Insulin, % (n)	100 (422)	66 (225)
GLP-1a, % (n)	< 1 (1)	30 (102)
OAD, % (n)	< 2 (6)	81 (275)
OAD + insulin, % (n)	< 2 (6)	47 (160)
Lipid lowering medication (statins), % (n)	47 (200)	85 (287)
Anti-hypertensive medication, % (n)	56 (238)	92(313)

*BMI* body mass index, *D-BP* diastolic blood pressure, *F* female, *GLP-1a* glucagon-like peptide 1 analogues, *HbA1c* glycated haemoglobin A1c, *HDL* cholesterol, high-density lipoprotein cholesterol, *LDL* cholesterol, low-density lipoprotein cholesterol, *OAD* oral antidiabetic drugs, *S-BP* systolic blood pressure, *T1D* type 1 diabetes, *T2D* type 2 diabetes

^a^Values are either presented as median (IQRs) or number (n) and percentages (%) of study population

**Fig. 1:**
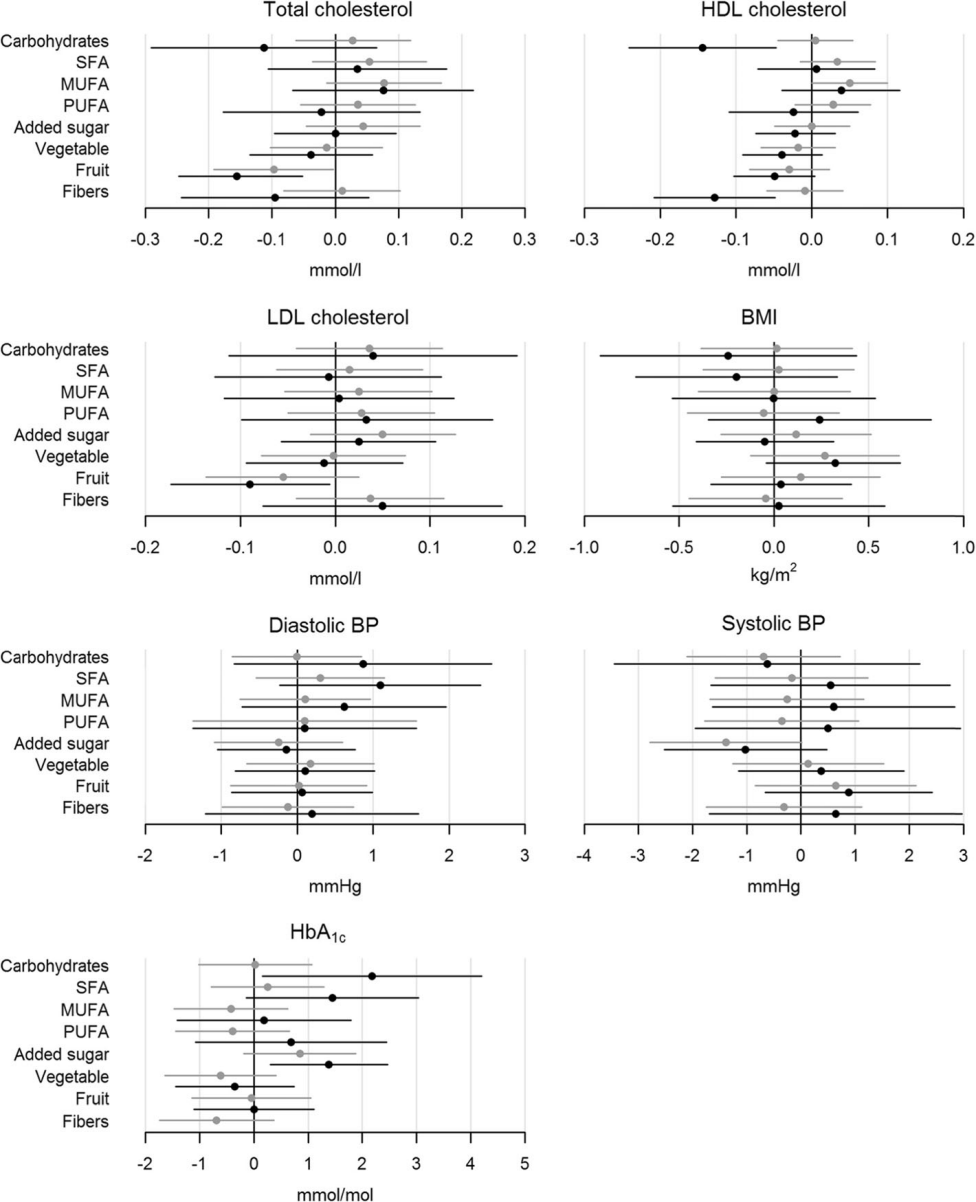
Shows the associations between the outcomes (total cholesterol, HDL cholesterol, LDL cholesterol, BMI, diastolic pressure, systolic blood pressure and HbA1c) and the eight chosen standardized food components (carbohydrates, SFA, MUFA, PUFA, added sugar, vegetables, fruit and dietary fibre) in persons with T1D. The grey line is the step-model 2 and the black line is the final step-model. Difference in outcomes with 95% CI are shown for a SD increase in the log-transformed food component. A difference is statistically significant when the 95% CI (horizontal lines) does not cross the vertical 0-line. BMI, body mass index; BP, blood pressure; HbA1c, glycated haemoglobin A1c; HDL cholesterol, high-density lipoprotein cholesterol; LDL cholesterol, low-density lipoprotein cholesterol; T1D, type 1 diabetes; SFA, saturated fatty acids; MUFA, monounsaturated fatty acids; PUFA, polyunsaturated fatty acids

**Fig. 2:**
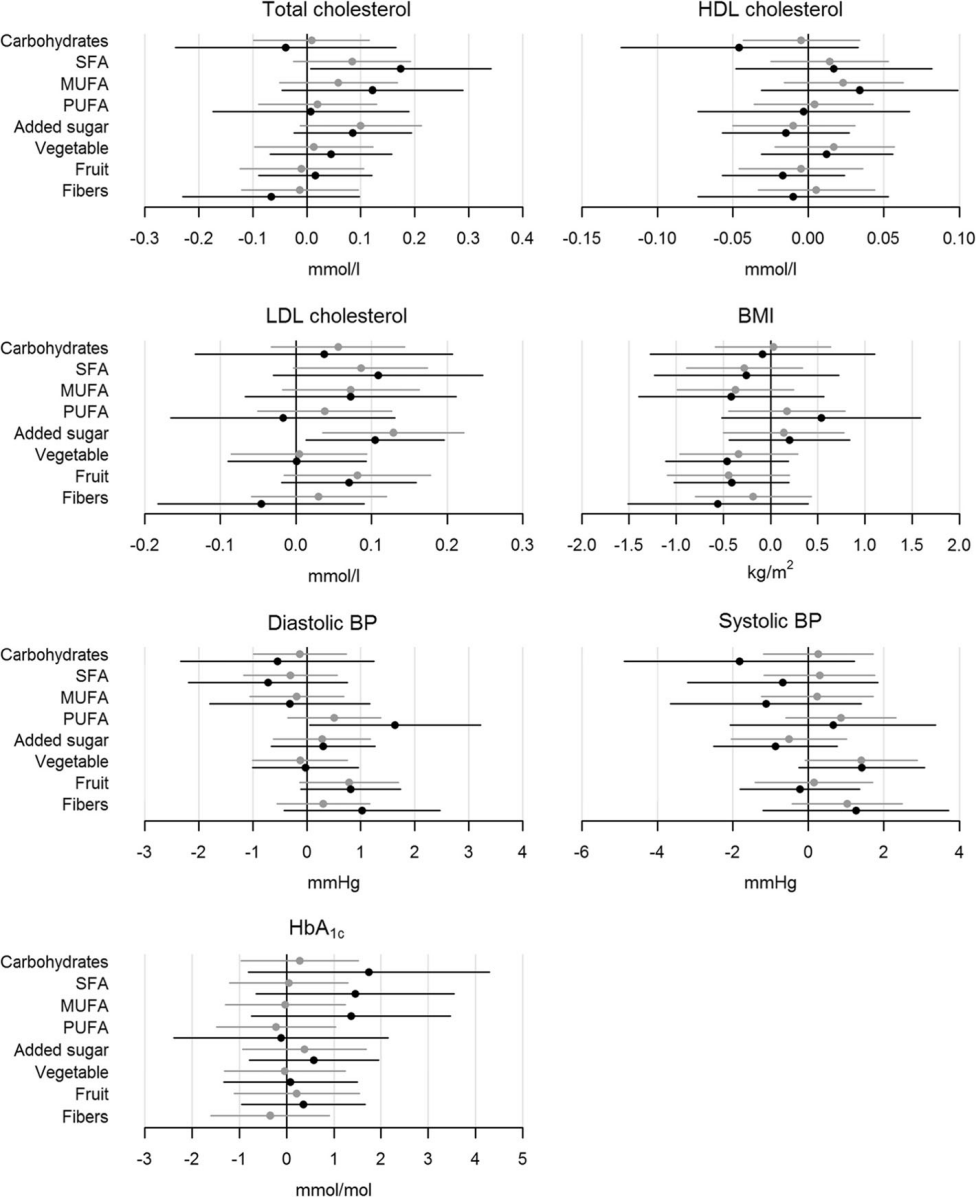
Shows the associations between the outcomes (total cholesterol, HDL cholesterol, LDL cholesterol, BMI, diastolic pressure, systolic blood pressure and HbA1c) and the eight chosen standardized food components (carbohydrates, SFA, MUFA, PUFA, added sugar, vegetables, fruit and dietary fibre) in persons with T2D. The grey line is the step-model 2 and the black line is the final step-model. Difference in outcomes with 95%-CI are shown for a SD increase in the log-transformed food component. A difference is statistically significant when the 95%-CI (horizontal lines) does not cross the vertical 0-line. BMI, body mass index; BP, blood pressure; HbA1c, glycated haemoglobin A1c; HDL cholesterol, high-density lipoprotein cholesterol; LDL cholesterol, low-density lipoprotein cholesterol; T2D, type 2 diabetes; SFA, saturated fatty acids; MUFA, monounsaturated fatty acids; PUFA, polyunsaturated fatty acids

In general, most food components were not associated with clinical outcomes. In persons with T1D, both higher intake of carbohydrates (*p* = 0.034) and higher intake of added sugar (*p* = 0.012) was associated with higher HbA1c, with the highest impact from carbohydrates (Fig. 1). Higher fruit intake was associated with lower levels of total C (*p* = 0.003) and LDL-C (*p* = 0.035) (Fig. 1). Both higher intake of carbohydrates (*p* = 0.004) and higher intake of dietary fibre (*p* = 0.002) was associated with lower HDL-C with similar impact (Fig. 1). In persons with T2D, higher SFA intake was associated with higher total C (*p* = 0.041), and higher intake of added sugar was associated with higher LDL-C (*p* = 0.025) (Fig. 2). Finally, higher PUFA intake was associated with higher DBP (Fig. 2).

## Discussion

The overall findings in this large-scale study in adults with T1D and T2D are in line with previous reports. Our findings highlight the importance of a continuous need to focus on improvement of diet quality and, in particular, on carbohydrate quantity and quality (i.e. intake of dietary fibre, added sugar and fruit) in order to improve metabolic control in people with diabetes.

One of the primary findings was that a higher intake of carbohydrates and added sugar was associated with an unfavourable lipid profile and HbA1c. However, the findings differed between diabetes classification, with the majority of significant dietary-related findings in the T1D group compared with the T2D group, even though the literature supports a similar focus on diet in both groups [1, 3, 5]. Persons with T1D who reported higher intake of food containing carbohydrates and added sugar also had higher HbA1c levels, which we did not find in persons with T2D, even though the dietary percentage of total energy deriving from carbohydrates was the same in both study groups (previously published data [7]). Even though the long-term effects of reducing the total carbohydrate intake are inconclusive, the impact of carbohydrates on both prandial and postprandial glucose excursions is well-known and of great importance in relation not only to the regulation of blood glucose levels and glycaemic control but also in relation to weight management. These factors have recently been investigated in several systematic reviews and metanalysis of randomized controlled trials (RTC) [5, 10–12].

In the current study, we found no relationship between HbA1c and intake of dietary fibre. Several other studies have examined the effect of dietary fibre on glycaemic control with varying results [13, 14, 20, 21]. A systematic review and meta-analysis of RCTs found that a high intake of dietary fibre reduced HbA1c by up to 6 mmol/mol (2.7%) in persons with T2D [20]. We found no association between fibre intake and HbA1c in our study. In general, the fibre intake was low (21–22 g/d) for the participants in this study compared with the recommended intake (> 40 g/d) [7].

We found that a higher fruit intake was related to a lower total C in T1D, and that a higher SFA intake was related to a higher total C in T2D. These differences may relate to some of the differences in dietary patterns and preferences between the two entities but may also mirror differences in Danish diabetes outpatient clinics. For example, in our clinic, persons with T1D are more frequently advised by a trained dietician than are those with T2D. In line with this, the intake of fruit in our study was also associated with a reduction in LDL-C in persons with T1D. Although limited literature is available on the effects of fruit intake on surrogate markers of CVD (e.g. lipid profile) in T1D, our findings support previous reports where fruit intake has been reported to be associated with an improved CVD profile [22]. One RCT found that a greater intake of fruit was associated with lower DBP [16] and a sub-analysis of fruit and vegetable intakes as part of the Mediterranean diet has also showed benefits in CVD prevention [22, 23]. It is well-known that a high intake of fruit (and vegetables) can be considered as a marker of a healthy lifestyle that coincides with more physical activity and less smoking, which is why it is difficult to separate the effects of fruit and vegetables from other factors related to a healthy lifestyle.

Added sugar was related to a higher LDL-C in persons with T2D and as outlined above, higher HbA1c in T1D. This underlines the importance of focusing on carbohydrate quality and avoiding “simple” carbohydrates. Other studies have found comparable results between intake of carbohydrates and HDL-C where a high-carbohydrate-high-fibre diet displayed greater reduction in HDL-C than did a low-carbohydrate-low-fibre diet in persons with T1D [15]. Knowledge is limited related to the association between sugar intake and LDL-C in persons with diabetes. However, in persons without diabetes, the intake of sugar is related to higher LDL-C concentration [24], which supports our findings.

A prospective cohort including 6192 persons with T1D and T2D found that higher intakes of dietary fibre were associated with and lower risk of CVD and lower mortality [25]. These hard endpoints are more powerful than our surrogate markers for CVD; however, our results clearly support these findings and are indicative of the importance in focusing on carbohydrate quality.

Our finding that intake of PUFA was associated with an increase in DBP in persons with T2D is in contrast to other studies, primary showing a reduction in blood pressure (both SBP and DBP) in relation to intake of PUFA [17, 26].The finding was surprising and may be a random finding without any clinical significance.

We found no relationship between BMI and diet in either group. This may be because the groups were relatively homogeneous within each group with a BMI range of 22–27 kg/m^2^ in persons with T1D and a BMI range of 26–33 kg/m^2^ in those with T2D, possibly indicating a well-known weakness, the non-response bias in survey questionnaires. Individuals participating in these types of survey are often more well-educated, more likely to have healthier eating habits and be more resourceful concerning diabetes self-management [7, 27]. In line with this, fewer was smokers, and more were better regulated in terms of HbA1c and total cholesterol and HDL-cholesterol compared with non-responders in our study. However, we tried to accommodate this by adjusting for level of education in our analyses. The dietary assessment was based on self-reported dietary intake using an FFQ with the risk of memory bias and social desirability bias leading individuals to under-report the consumption of unhealthy foods, for example, foods high in added sugar due to the focus on avoiding sugar in diabetes [27–29] . This may have resulted in under-reporting in the study, thereby influencing our study results. Dietary under-reporting is a frequent problem in persons with T2D, an aspect we have previously addressed [7], and to reduce the risk of memory bias, we used an FFQ with a time span of 3 months. The FFQ used for dietary data collection in our study has previously been validated in persons with T1D and T2D [9] and was found to be a reliable tool for assessment of dietary intake, especially intake of foods containing carbohydrates, dietary fibre and added sugar.

Another potential weakness in our study is the time point for extracting clinical data from the EMR. Data were not measured on the day the questionnaire was completed. Furthermore, lack of compliance with the prescribed medicine in diabetes is known and is a factor we cannot adjust for [30]. Some of our findings are somewhat in contrast to other published results and indeed difficult to explain and therefore deserves attention in future research in similar patient populations.

## Conclusion

In conclusion, we found that the total intake and quality of carbohydrates as well as the fat quality were associated with HbA1c and plasma lipids in Danish adults with T1D and T2D. Our findings emphasise the importance of constant focus on dietary support by dietitians to improve the diet quality in order to obtain metabolic control and possibly reduce cardiovascular risk.

## Data Availability

Contact the corresponding author bettina.ewers@regionh.dk for access to data or materials.

## Abbreviations

T1D: Type 1 diabetes
T2D: Type 2 diabetes
HbA1c: Glycated haemoglobin A1c
BMI: Body mass index
CVD: Cardiovascular disease
EMR: Electronic Medical Record
SFA: Saturated fatty acids
MUFA: Monounsaturated fatty acids
PUFA: Polyunsaturated fatty acids
FFQ: Food frequency questionnaire
Total C: Total cholesterol
HDL-C: High-density lipoprotein cholesterol
LDL-C: Low-density lipoprotein cholesterol
DBP: Diastolic blood pressure
SBP: Systolic blood pressure
OAD: Oral antidiabetic medications
IQR: Interquartile range
SD: Standard deviation
GLP-1a: Glucagon-like peptide-1 receptor analogues
RCT: Randomized controlled trials

## References

[1] Mann JI, De Leeuw I, Hermansen K, Karamanos B, Karlstrom B, Katsilambros N (2004). Evidence-based nutritional approaches to the treatment and prevention of diabetes mellitus. Nutr Metab Cardiovasc Dis.

[2] Mann JI (2006). Nutrition recommendations for the treatment and prevention of type 2 diabetes and the metabolic syndrome: an evidenced-based review. Nutr Rev.

[3] Evert AB, Boucher JL, Cypress M, Dunbar SA, Franz MJ, Mayer-Davis EJ (2014). Nutrition Therapy Recommendations for the Management of Adults With Diabetes. Diabetes Care.

[4] Davies MJ, D'Alessio DA, Fradkin J, Kernan WN, Mathieu C, Mingrone G, et al. Management of Hyperglycemia in Type 2 Diabetes, 2018. A consensus report by the American Diabetes Association (ADA) and the European Association for the Study of diabetes (EASD). Diabetes Care. 2018;41(12):2669–701. 10.2337/dci18-0033 PMC6245208 30291106

[5] Wheeler ML, Dunbar SA, Jaacks LM, Karmally W, Mayer-Davis EJ, Wylie-Rosett J (2012). Macronutrients, food groups, and eating patterns in the Management of Diabetes. Diabetes Care.

[6] Vitale M, Masulli M, Cocozza S, Anichini R, Babini AC, Boemi M (2016). Sex differences in food choices, adherence to dietary recommendations and plasma lipid profile in type 2 diabetes - the TOSCA.IT study. Nutr Metab Cardiovasc Dis.

[7] Ewers B, Trolle E, Jacobsen SS, Vististen D, Almdal TP, Vilsboll T (2019). Dietary habits and adherence to dietary recommendations in patients with type 1 and type 2 diabetes compared with the general population in Denmark. Nutrition.

[8] Franz MJ, MacLeod J, Evert A, Brown C, Gradwell E, Handu D (2017). Academy of nutrition and dietetics nutrition practice guideline for type 1 and type 2 diabetes in adults: systematic review of evidence for medical nutrition therapy effectiveness and recommendations for integration into the nutrition care process. J Acad Nutr Diet.

[9] Bentzen SMR, Knudsen VK, Christiensen T, Ewers B (2016). Relative validity of a web-based food frequency questionnaire for patients with type 1 and type 2 diabetes in Denmark. Nutr Diabetes.

[10] Nielsen JV, Gando C, Joensson E, Paulsson C (2012). Low carbohydrate diet in type 1 diabetes, long-term improvement and adherence: A clinical audit. Diabetol Metab Syndr.

[11] Snorgaard O, Poulsen GM, Andersen HK, Astrup A (2017). Systematic review and meta-analysis of dietary carbohydrate restriction in patients with type 2 diabetes. BMJ Open Diabetes Res Care.

[12] Sainsbury E, Kizirian NV, Partridge SR, Gill T, Colagiuri S, Gibson AA (2018). Effect of dietary carbohydrate restriction on glycemic control in adults with diabetes: a systematic review and meta-analysis. Diabetes Res Clin Pract.

[13] Post RE, Mainous AG, King DE, Simpson KN (2012). Dietary fiber for the treatment of type 2 diabetes mellitus: a meta-analysis. J Am Board Fam Med.

[14] Velazquez-Lopez L, Munoz-Torres AV, Garcia-Pena C, Lopez-Alarcon M, Islas-Andrade S, Escobedo-de la Pena J (2016). Fiber in Diet Is Associated with Improvement of Glycated Hemoglobin and Lipid Profile in Mexican Patients with Type 2 Diabetes. J Diabetes Res.

[15] Anderson JW, Zeigler JA, Deakins DA, Floore TL, Dillon DW, Wood CL (1991). Metabolic effects of high-carbohydrate, high-fiber diets for insulin-dependent diabetic individuals. Am J Clin Nutr.

[16] Sanjeevi N, Lipsky LM, Nansel TR (2018). Cardiovascular biomarkers in association with dietary intake in a longitudinal study of youth with type 1 diabetes. Nutrients..

[17] Saneei P, Salehi-Abargouei A, Esmaillzadeh A, Azadbakht L (2014). Influence of dietary approaches to stop hypertension (DASH) diet on blood pressure: a systematic review and meta-analysis on randomized controlled trials. Nutr Metab Cardiovasc Dis.

[18] American Diabetes A (2018). Lifestyle management: standards of medical Care in Diabetes-2018. Diabetes Care.

[19] Rostnthal R, Cooper H, Hedges LV (1994). Parametric measures of effect size. The handbook of research synthesis.

[20] Silva FM, Kramer CK, de Almeida JC, Steemburgo T, Gross JL, Azevedo MJ (2013). Fiber intake and glycemic control in patients with type 2 diabetes mellitus: a systematic review with meta-analysis of randomized controlled trials. Nutr Rev.

[21] Cugnet-Anceau C, Nazare JA, Biorklund M, Le Coquil E, Sassolas A, Sothier M (2010). A controlled study of consumption of beta-glucan-enriched soups for 2 months by type 2 diabetic free-living subjects. Br J Nutr.

[22] Widmer RJ, Flammer AJ, Lerman LO, Lerman A (2015). The Mediterranean diet, its components, and cardiovascular disease. Am J Med.

[23] Georgoulis M, Kontogianni MD, Yiannakouris N (2014). Mediterranean diet and diabetes: prevention and treatment. Nutrients..

[24] Te Morenga LA, Howatson AJ, Jones RM, Mann J (2014). Dietary sugars and cardiometabolic risk: systematic review and meta-analyses of randomized controlled trials of the effects on blood pressure and lipids. Am J Clin Nutr.

[25] Burger KN, Beulens JW, van der Schouw YT, Sluijs I, Spijkerman AM, Sluik D (2012). Dietary fiber, carbohydrate quality and quantity, and mortality risk of individuals with diabetes mellitus. PLoS One.

[26] Shantakumari N, Eldeeb RA, Ibrahim SA, Sreedharan J, Otoum S (2014). Effect of PUFA on patients with hypertension: a hospital based study. Indian Heart J.

[27] Sallé A, Ryan M, Ritz P (2006). Underreporting of food intake in obese diabetic and nondiabetic patients. Diabetes Care.

[28] Castro-Quezada I, Ruano-Rodriguez C, Ribas-Barba L, Serra-Majem L (2015). Misreporting in nutritional surveys: methodological implications. Nutr Hosp.

[29] Maurer J, Taren DL, Teixeira PJ, Thomson CA, Lohman TG, Going SB (2006). The psychosocial and behavioral characteristics related to energy misreporting. Nutr Rev.

[30] Jensen ML, Jorgensen ME, Hansen EH, Aagaard L, Carstensen B (2017). Long-term patterns of adherence to medication therapy among patients with type 2 diabetes mellitus in Denmark: the importance of initiation. PLoS One.

